# Maternal Caffeine Consumption and Its Impact on the Fetus: A Review

**DOI:** 10.7759/cureus.48266

**Published:** 2023-11-04

**Authors:** Hunter Lakin, Patrick Sheehan, Varun Soti

**Affiliations:** 1 Pediatrics, Lake Erie College of Osteopathic Medicine, Elmira, USA; 2 Internal Medicine, Lake Erie College of Osteopathic Medicine, Elmira, USA; 3 Pharmacology and Therapeutics, Lake Erie College of Osteopathic Medicine, Elmira, USA

**Keywords:** birth weight, fetal breathing rate, fetal heart rate, pregnancy-related issues, caffeine consumption

## Abstract

Maintaining a healthy diet is essential for pregnant women and their developing fetuses, including being mindful of caffeine consumption. While consuming caffeine during pregnancy is generally safe, there is a concern among healthcare practitioners about whether it can adversely impact pregnancy. There is a lack of accurate information about the effects of caffeine on fetal development and inadequate education on the risks of excessive caffeine intake during pregnancy. Therefore, to address this gap, our review provides an overview of the current literature on the impact of caffeine consumption during pregnancy on fetal development. We thoroughly searched databases, including PubMed and Clinicatrial.gov, from September 2022 to January 2023, focusing on relevant clinical studies with a level of clinical evidence II or higher. Our findings reveal that caffeine intake during pregnancy has notable effects on human fetal development. It increases fetal breathing and heart rates but can lead to reduced growth and a lower birth weight. Although it does not affect gestational length or cause hypertension, caffeine increases uterine contractions, potentially resulting in spontaneous abortion. In some cases, it even contributes to the development of pre-eclampsia in the later stages of pregnancy. However, the data on the association between caffeine consumption and the risk of congenital disabilities remains inconclusive. Based on these findings, it is clear that more extensive research is needed to fully understand the impact of caffeine consumption on the development of congenital disabilities in infants born to caffeine-consuming pregnant women. Furthermore, gaining a deeper understanding of how caffeine affects fetal development and pregnancy mechanisms is crucial.

## Introduction and background

Pregnancy is a dynamic process in which a woman’s body constantly works to protect itself and the growing fetus. Many internal and external factors, including a woman’s diet, contribute to the fetus’s well-being [1]. Consequently, obstetricians-gynecologists recommend that pregnant women regulate their caffeine intake [2]. Caffeine is a natural component found in coffee, cacao, and guarana plants, present in their fruits, leaves, and beans. It is also added to beverages and supplements. Caffeine can be consumed in multiple forms, including coffee, espresso, tea, soda, dark chocolate, and breakfast cereals [3].

For instance, a cup of brewed coffee, which amounts to eight ounces, contains approximately 95 milligrams (mg) of caffeine. Instant coffee, in the same quantity, has around 60 mg of caffeine. Decaffeinated coffee, on the other hand, contains roughly 4 mg of caffeine. Moving to espresso, one shot, equating to 1.5 ounces, contains about 65 mg of caffeine. Black tea, in a cup, contains about 47 mg of caffeine, whereas green tea has around 28 mg. As for soda, a 12-ounce can of either regular or diet dark cola contains about 40 mg of caffeine. The same quantity of Mountain Dew contains 55 mg of caffeine. Moving on to chocolate, one ounce of dark chocolate has about 24 mg of caffeine, while milk chocolate contains one-quarter of that amount. Guarana, a seed extracted from a South American plant and processed for use in various foods, energy drinks, and supplements, contains approximately four times the amount of caffeine found in coffee beans. Some beverages that incorporate extracts from these seeds can contain up to 125 mg of caffeine per serving. Even an eight-ounce energy drink contains about 85 mg of caffeine. However, it is important to note that the standard serving size for energy drinks is 16 ounces, effectively doubling the caffeine content to 170 mg. Energy shots, on the other hand, are significantly more concentrated, with a small two-ounce shot containing around 200 mg of caffeine [3].

It is crucial to exercise caution when consuming excessive amounts of caffeinated beverages, such as soda and energy drinks, as they are often served chilled. Chilled caffeinated beverages can usually be consumed in large quantities. If a pregnant woman consumes large amounts of chilled caffeinated products, it puts a more significant risk on the fetus with increased exposure. Therefore, the American College of Obstetricians and Gynecologists suggests that pregnant women should consume no more than 200 mg of caffeine per day, equivalent to two cups of coffee [4]. Adhering to these guidelines can help prevent caffeine’s potential negative impacts on pregnancy and fetal development. These negative impacts may include restricted fetal growth, low birth weight, fetal malformation, premature birth, miscarriage, and spontaneous abortion [5-7].

Despite these guidelines, studies have shown that about 70% of pregnant women still consume caffeine. Some even consume quantities between 300 and 500 mg per day during pregnancy. Caffeine’s impact on the fetus, suboptimal education regarding excessive caffeine intake, and insufficient research may contribute to this trend. Furthermore, healthcare providers may show reluctance to enforce guidelines, while pregnant women may opt not to comply [8].

This paper aims to provide an overview of existing literature on caffeine consumption during pregnancy and the impact of caffeine on fetal development in different aspects of gestational health. It intends to bridge the gap in current research and identify the research areas that medical practitioners and pregnant women should consider. By doing so, both clinicians and expectant mothers can make informed decisions.

## Review

Literature search and study selection

**Figure 1:**
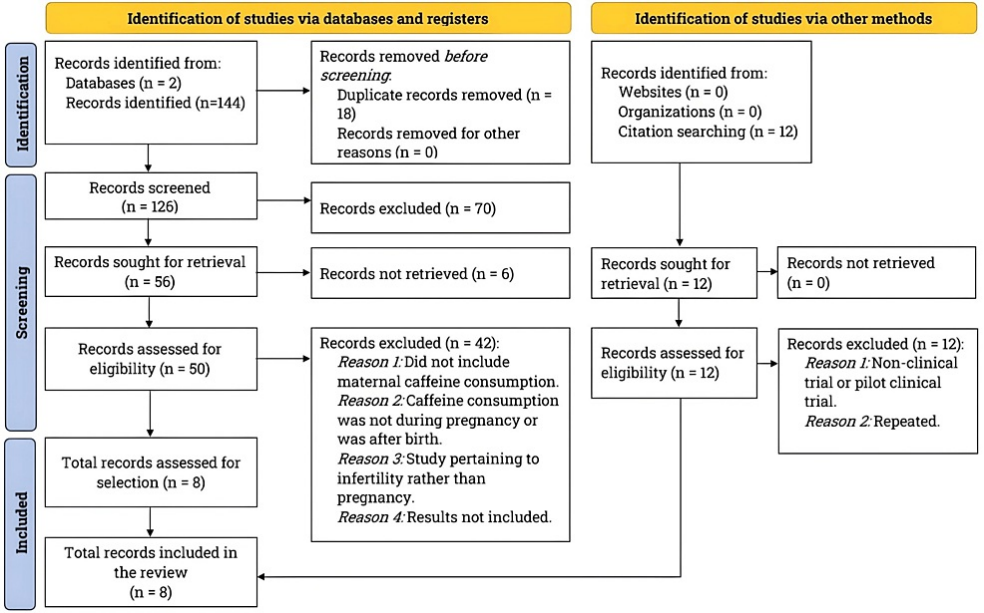
Literature search and study selection The diagram displays the steps involved in searching the literature and selecting studies. We conducted a thorough literature search using PubMed and ClinicalTrials.gov, following the guidelines outlined by PRISMA. Our focus was on clinical studies examining the effects of maternal caffeine consumption during pregnancy on fetal development and pregnancy outcomes.

We conducted a literature search from September 2022 to January 2023, following the Preferred Reporting Items for Systematic Reviews and Meta-Analyses (PRISMA) guidelines [9]. We used two databases, PubMed and Clinicaltrials.gov, to search for studies on the topics of “Caffeine and Fetal Growth” (30 studies) and “Pregnancy and Caffeine” (45 studies) on PubMed. On Clinicaltrials.gov, the search term “Caffeine Consumption During Pregnancy” yielded four studies, while “Pregnancy and Caffeine” retrieved 35 articles. Please refer to the PRISMA flowchart in Figure 1 for our literature search and study selection process details.

We selected relevant studies in English, following our inclusion and exclusion criteria (refer to Table 1). Moreover, we assessed each study for the level of clinical evidence based on the existing literature [10] and only included studies with clinical evidence II or higher levels.

**Table 1:** Criteria for study selection This review encompassed studies published in English with clinical evidence of level II or higher that satisfied the inclusion criteria outlined in this table. Specifically, it focused on studies investigating the effects of caffeine consumption on fetal development and pregnancy outcomes in pregnant women.

Inclusion criteria	Exclusion criteria
Randomized controlled trial	Pilot clinical studies
Non-randomized controlled trial	Case series
Single-blinded studies	Case reports
Double-blinded studies	Reviews
Case-controlled studies	Metanalyses
Crossover studies	Pre-clinical studies
Population-based studies	Systematic reviews
Observational studies	Commentaries

Understanding caffeine: how it works, its impact on the body, and potential risks

Caffeine consumption has widespread effects on the body, from metabolism to biological impacts and potential adverse outcomes. It is the most widely consumed CNS stimulant worldwide, from coffee to tea, chocolate, and soda. After crossing the blood-brain barrier, caffeine acts primarily on the brain’s adenosine receptors. Adenosine reduces activity in the ascending arousal system, diminishing the amount of dopamine in the brain. However, caffeine blocks the adenosine-mediated depression of the arousal system by inhibiting adenosine receptors A1 and A2A, thus producing a stimulant effect [11].

Additionally, caffeine increases dopamine receptors D2 and D3 in the putamen and ventral striatum, further contributing to greater alertness. However, this mechanism also causes people to develop caffeine dependence, eventually leading to an addiction to caffeinated products. Caffeine improves response time and alertness, explaining why people tend to increase intake when sleep-deprived [11].

Adenosine receptors are present not only in the CNS but also in other parts of the body. Caffeine antagonizes adenosine receptors, which causes a surge of catecholamines and an altered vascular tone, resulting in a rise in systolic blood pressure. This increased catecholamines can lead to increased fetal heart rate and placental vasoconstriction, leading to decreased fetal oxygenation in utero. Furthermore, caffeine stimulates respiratory drive through increased medullary response, augments gastrointestinal motility, and acts as a diuretic by increasing glomerular filtration and blood flow [11-12].

Caffeine’s lipophilic nature enables it to cross the blood-brain barrier in adults. However, it also traverses the placental barrier, leading to comparable caffeine levels in both the fetus and mother [12]. Even though the fetus and placenta have a limited capacity to metabolize caffeine, the blood-placental barrier is permeable to caffeine. The latter can remain in the uterine fluid for prolonged periods, increasing its impact on the fetus. While the mother has a minimal first-pass effort at metabolizing caffeine, the developing fetus experiences the full effects of the ingested caffeine. Variations in caffeine metabolism exist, and some studies indicate that consuming as low as 100 to 200 mg of caffeine daily can prompt pregnancy complications. Common side effects include subfertility, intrauterine growth retardation, low birth weight, and spontaneous abortion [13].

The process of caffeine absorption takes place at a rapid pace in the small intestine. After approximately 30 minutes, there is a marked rise in the stimulatory effect, but the liver contributes toward its metabolism. The cytochrome P 450 (CYP) 1A2 enzyme is the primary contributor, with metabolites such as paraxanthine, theobromine, and theophylline produced upon demethylation by CYP1A2. Further acetylation creates even more metabolites, such as 1-methyl-uric acid, 5-acetylamino-6-formylamino-3-methyluracil, 1-methylxanthine, 1,7-dimethyl-uric acid, and 1,7-dimethylxanthine, which are excreted into the urine [14].

The typical half-life of caffeine is generally five hours. However, cigarette smoking reduces its half-life, while pregnant women experience an extended half-life. Pregnant women may have a caffeine half-life of up to 15 hours in the final trimester. Newborns and premature infants have an even more enhanced half-life of caffeine, reaching up to eight and 100 hours, respectively, attributed to the immaturity of the CYP system [11]. Most infants excrete more caffeine through the kidneys, and unlike adults, much more is excreted unchanged [12].

Caffeine consumption has adverse effects ranging from mild, such as restlessness, increased urination, anxiety, insomnia, and irritability, to severe outcomes, such as seizure, arrhythmia, altered mental status and visual perception, and rhabdomyolysis from excessive consumption (more than 200 mg/day) [4,11]. Risks of withdrawal exist for high-caffeine consumers. Though caffeine consumption has no absolute contraindications, its physiological effects on the body’s different organs and various pathological conditions may warrant regulation. Some critical conditions that necessitate caution in caffeine intake include cardiovascular disease, anxiety, peptic ulcer disease, decreased renal function, reduced hepatic function, seizures, and pregnancy [11].

Exploring the impact of caffeine: unraveling its influence on fetal development

Salvador and Koos (1989) conducted a single-blind crossover study with eight patients to examine the effects of caffeinated and decaffeinated coffee on fetal breathing. The study included pregnant women (32-36 weeks of gestation) who were non-smokers. Four women served as the control group and consumed decaffeinated coffee, while the experimental group consisted of four women who consumed caffeinated coffee. The researchers monitored fetal breathing rate, fetal heart rate, and maternal epinephrine levels after consuming either type of coffee. They used ultrasound to track fetal chest and abdominal movements and Doppler to monitor heart rate. Moreover, the study protocol required sampling maternal blood before and after 30 minutes of coffee consumption in the control and experimental groups [15].

The study findings showed that maternal consumption of caffeinated coffee led to a 2.3-fold increase in epinephrine levels compared to decaffeinated coffee (p<0.05). After 30 minutes of caffeine consumption, epinephrine levels rose from 200 picograms per milliliter (pg/mL) to 455 pg/mL. The fetal breathing rate significantly increased following maternal caffeine consumption (p<0.01). This increase in fetal breathing could potentially result from the biological effects of caffeine on the body. It is critical to underline that caffeine’s vasoconstricting properties can reduce blood flow to the fetus, causing the fetus to compensate by increasing its breathing rate [15].

Furthermore, the study demonstrated that increased maternal epinephrine levels after caffeinated coffee consumption contributed to the elevated fetal breathing rate. Although the study’s sample size could have been larger, the blinded study observer prevented potential bias by remaining unaware of whether the mother consumed caffeinated or decaffeinated coffee. These findings provide direct evidence of caffeine’s impact on fetal activity and physiology during pregnancy. However, studies with extended sample sizes must be conducted to further elaborate on this evidence [15].

Mulder et al. (2009) conducted a non-randomized controlled trial to understand further how maternal caffeine intake affects fetal response. Thirteen healthy pregnant women, with a mean age of 29 years, participated in the study. These non-obese females had unremarkable obstetric histories. Additionally, they had no history of smoking, alcohol consumption, or any medication or drug use. Participants were required to keep a diary of their food and caffeine consumption for one week before the start of the study. Caffeine sources included coffee, tea, cola, and dark chocolate. The control group consisted of pregnant women under “normal conditions.” The study recorded caffeine consumption and utilized ultrasound to monitor changes in the fetal heart rate, general movements, general breathing movements, and behavioral state [16].

The findings revealed that maternal caffeine consumption significantly increased the fetuses’ awake time (p<0.01). In addition, there was a significant increase in the number of fetal heart rate accelerations (p<0.05) and general fetal movements (p<0.05). However, there was no conclusive evidence regarding the impact of maternal caffeine consumption on the fetal breathing rate. It is worth noting that the researchers did not precisely define “normal conditions” for the control group, and the study had a smaller sample size. Nonetheless, these results demonstrate that maternal caffeine consumption alters fetal functionality, indicating its impact on the fetus’s environment [16].

Building on the remarkable findings of Mulder et al. (2009), in a breakthrough crossover clinical trial, Buscicchio et al. (2012) recruited 50 patients to investigate the impact of maternal caffeine and chocolate intake on fetal heart rate. The study patients were pregnant women with uncomplicated pregnancies and recorded fetal heart rates before and 60 minutes after consuming espresso or dark chocolate. The researchers used computerized fetal heart rate recording and cardiotocography to collect data. They also examined uterine contraction peaks, the number of small and large accelerations, and the duration of episodes of high variation and short-term variation to better understand caffeine’s effects on the fetal heart [17].

The study findings indicated a significant increase in fetal heart rate accelerations (p<0.001), fetal heart rate variability (p<0.001), and the frequency of uterine contractions (p<0.001) following both espresso and chocolate consumption, suggesting a stimulating effect of caffeine. Notably, this study stands out as it thoroughly investigated the impact of caffeine on fetal heart rate and explored various aspects of fetal activity using computerized cardiotocography, providing a comprehensive understanding of caffeine’s effects on fetal functionality. These findings are significant as they reveal potential mechanisms by which increased fetal heart rate and accelerated uterine contractions induced by caffeine could contribute to miscarriage or spontaneous abortion [17].

In a different prospective, population-based study involving 1,724 Caucasian pregnant women, Cook et al. (1997) examined the impact of caffeine consumption on fetal growth during pregnancy. Of the 640 pregnant women who completed the study, 500 were non-smokers and 140 were smokers. The study protocol involved monitoring the blood levels of caffeine and cotinine, a marker for cigarette smoking. A blood cotinine level of 15 mg/mL was used as the threshold to distinguish smokers from non-smokers. The researchers interviewed the patients at the beginning, 28th, and 36th weeks of gestation. The patients self-reported their caffeine intake, including the cups of coffee, tea, cocoa, and cola consumed each week [18].

The study findings revealed increased blood caffeine levels from 2.35 micrograms per liter (µg/L) at the initial encounter to 4.12 µg/L at 36 weeks of gestation. The researchers concluded caffeine intake was negatively correlated with fetal birth weight (p<0.001). These findings support the hypothesis that caffeine consumption during pregnancy is associated with intrauterine growth restriction. However, it is essential to note that this study had some limitations. Firstly, it failed to adequately control the confounding effects of smoking, which could have influenced fetal growth in ways not accounted for by the researchers. Therefore, further research must be conducted with proper control groups and limited confounding factors to more effectively assess how caffeine consumption can impact fetal growth. Secondly, a significant portion of the data used in this study relied on patient interviews, which introduced the possibility of bias or false reporting. Despite its limitations, one major strength of this study was its large sample size compared to others, providing crucial evidence on how caffeine consumption during pregnancy can potentially have adverse effects on fetal growth and development [18].

A decade later, in an extensive, double-blind, randomized controlled trial involving 1,207 patients, Bech et al. (2007) further explored the impact of reducing maternal caffeine intake on fetal birth weight and length of gestation. The researchers enlisted Danish women who were less than 20 weeks pregnant and consumed at least three cups of coffee daily. These women had no notable medical history of conditions that could affect pregnancy outcomes, such as the low birth weight of previous children, preterm birth, kidney disease, epilepsy, diabetes, or metabolic disorders [19].

For the study, the researchers randomly divided participants into two groups: one received caffeinated instant coffee (568 pregnant women), while the other received decaffeinated instant coffee (629 pregnant women). They purchased coffee in identical, unlabeled boxes and sent six boxes to each participant, with a unique serial number assigned to each box. The women could request as much coffee as they needed. The participants replaced their regular coffee with the provided instant coffee as part of the study protocol. However, they did not receive any instructions to avoid other sources of caffeine, such as tea, cocoa, cola, or coffee offered by others. The researchers interviewed the participants at gestational weeks 20, 25, and 34, and four weeks after the expected delivery date, to gather data on their daily consumption of the study coffee, other caffeinated beverages, and smoking habits. The primary outcomes measured were birth weight and length of gestation [19].

The study findings indicated no significant differences in the average birth weight or length of gestation between women in the decaffeinated coffee group and those in the caffeinated coffee group. However, after adjusting for gestation length, parity, pre-pregnancy body mass index, and smoking status at the start of the study, the researchers observed that the mean birth weight of babies born to women in the caffeinated group was 16 grams lower than that of those born to women in the decaffeinated group (p<0.05). It is important to note that there were no long-term follow-ups regarding neonatal growth rates. Nonetheless, these findings underscore the significant negative impact of caffeine consumption on newborn birth weight [19].

In a groundbreaking parallel, double-blind, randomized controlled trial, Mogollon et al. (2013) investigated the impact of flavanol-rich chocolate on blood pressure, endothelial function, and the occurrence of pre-eclampsia in healthy pregnant patients. Flavanols in dark chocolate are antioxidants that promote nitric oxide-mediated vasodilation. Thus, flavanols can influence endothelial function, reducing blood pressure. The researchers recruited 44 pregnant women and randomly divided them into two groups: 23 women consumed high-flavanol chocolates, while 21 women consumed low-flavanol chocolates. All participants were non-smokers with no underlying health issues. Pregnant women with chronic hypertension, renal dysfunction, hypertension medication, and a family history of premature cardiovascular disease were excluded from the study [20].

At specific time intervals (0, 60, 120, and 180 minutes after a single 40-gram dose of chocolate), as well as 6 and 12 weeks after a daily intake of 20 grams of chocolate, the researchers measured plasma concentrations of flavonols (epicatechin) and theobromine. Additionally, they measured flow-mediated dilation and blood pressure by assessing the brachial artery through ultrasound and electronic monitors. The study results demonstrated a significant increase (p<0.001) in plasma epicatechin levels 180 minutes after consuming 40 grams of high-flavanol chocolate compared to low-flavanol chocolate. Theobromine concentrations were significantly higher at 180 minutes and 12 weeks after consuming the low-flavanol chocolate (p<0.001). Flow-mediated dilation did not differ between the two groups at any pre-defined time point [20].

A noteworthy observation from the study was the absence of any hypertension or pre-eclampsia-related complications in pregnant women who consumed high-flavanol chocolate. On the other hand, participants in the low-flavanol chocolate group experienced pre-eclampsia in the later stages of pregnancy. Pre-eclampsia poses a significant risk and can increase the chances of perinatal mortality. These findings suggest that maternal caffeine consumption, in conjunction with low levels of flavonols and high levels of caffeine in chocolate, might contribute to the risk of pre-eclampsia and harm to the fetus [20].

There have been concerns among healthcare pediatricians and pregnant women about caffeine’s involvement in developing congenital disabilities. Henceforth, Rosenberg et al. (1982), in a comprehensive, non-randomized, case-controlled study, enrolled 2,030 patients and examined the prevalence of selected congenital disabilities concerning caffeine-containing beverages. They divided the infants into two groups. The first group consisted of infants with inguinal hernias, cleft lips (with or without cleft palate), isolated cleft palate, cardiac defects (excluding heart murmur), pyloric stenosis, or neural tube defects. The second group served as the control and included infants with all other malformations. Each of these case groups comprised 100 or more affected infants. In the study, infants’ mothers self-reported caffeine consumption during pregnancy [21].

This study found no significant difference between the case and control infants (p>0.05). While the numerical results were not statistically significant, they provide evidence that caffeine consumption during pregnancy is less likely to lead to specific birth defects, as mentioned earlier. However, there are notable limitations in this study. First, mothers were only asked about their caffeine consumption after childbirth, potentially leading to inaccurate reporting. Additionally, caffeine intake was self-reported based on the number of cups consumed, which introduces variations in the definition of a “cup” and the strength of caffeine among participants. Despite these drawbacks, the study considered confounding factors, such as technical variables, maternal characteristics, and maternal diseases, to maximize result accuracy. Furthermore, the study’s large sample size and careful design support the analysis of possible congenital disabilities associated with maternal caffeine consumption [21].

In an attempt to further expand on the research endeavors of Rosenberg et al. (1982) and find more specific and conclusive evidence, Browne et al. (2007) examined the association between maternal caffeine consumption and the risk of cardiovascular malformations in a large, case-controlled study involving 8,153 participants. This sample contained 4,196 infants with cardiovascular malformations, and 3,957 infants served as the control. The study utilized congenital disability surveillance registries across eight states (Arkansas, California, Georgia, Iowa, Massachusetts, New Jersey, New York, and Texas) to recruit participants. The researchers excluded women with multiple gestations, a history of insulin use, or gestational diabetes. They conducted interviews by telephone to collect information about demographic characteristics, pregnancy history, and caffeine exposures before and during pregnancy. Specifically, they asked participants about their typical intake of caffeinated coffee, tea, soda, soft drinks, and chocolate in the year leading up to and during pregnancy [22].

The study findings suggested that moderate caffeine consumption (less than 300 mg/day) increased the risks of tetralogy of Fallot, d-transposition of the great arteries, and cardiovascular malformations with multiple congenital anomalies in non-smokers. However, it is essential to note that these results were not statistically significant and could have been due to several study limitations. Firstly, the study did not account for the variation in coffee or tea brewing strength, which can significantly influence the outcomes. Secondly, some cardiovascular malformations have the potential to resolve with time, and the study did not consider this aspect. Therefore, it might affect the study results [22].

Another confounding factor is the lack of documentation regarding the specific period of coffee or other caffeinated beverage consumption before and during pregnancy. Finally, a potential recall bias could impact the statistical analysis of the study results. Therefore, further research addressing these limitations is necessary to fully understand the significant impact of caffeine on cardiovascular malformations [22]. Please refer to Table 2, which illustrates the key findings of the reviewed study revealing the effects of caffeine consumption on fetal development and pregnancy.

**Table 2:** Key study findings: the impact of maternal caffeine consumption on fetus The table provides crucial insights from the reviewed studies, including their design, number of patients, and significant findings. These studies demonstrate the impact of caffeine on various aspects of fetal development, such as fetal growth rate, fetal heart rate, fetal breathing rate, congenital malformations, and pregnancy-related complications. p, probability value.

Researchers	Study design	Sample size	Effects on maternal caffeine consumption on fetus and pregnancy	Statistical significance
Salvador and Koos (1989) [15]	Single-blind crossover study	8	Increased fetal breathing rate	p<0.01
Mulder et al. (2009) [16]	Non-randomized controlled trial	13	Increased fetal awake time, increased fetal heart rate accelerations, and no effect on fetal breathing rate	p<0.01 and p<0.05
Buscicchio et al. (2012) [17]	Crossover clinical trial	50	Increased fetal heart rate, increased fetal heart rate variability, and increased uterine contractions	p<0.001, p<0.001, and p<0.001
Cook et al. (1997) [18]	Prospective, population-based study	640	Decreased birth weight	p<0.001
Bech et al. (2007) [19]	Double-blind, randomized controlled trial	1,207	Decreased birth weight. No effect on gestation length	p<0.05
Mogollon et al. (2013) [20]	Parallel, double-blind, randomized controlled trial	44	Low-flavanol-rich chocolate resulted in pre-eclampsia in the later stages of pregnancy. No hypertension	Not reported
Rosenberg et al. (1982) [21]	Non-randomized, case-controlled trial	2,030	No effect on the development of congenital birth defects	No statistical difference between infants born to caffeine and non-caffeine-drinking pregnant study participants
Browne et al. (2007) [22]	Prospective, population-based study	8,153	Increased risk of cardiovascular malformations in non-smoker caffeine drinkers	Not statistically significant

## Conclusions

Pregnancy is a critical period for both the woman and her fetus. To ensure a healthy pregnancy, healthcare providers strongly advise against using certain medications and consuming specific types of food, alcohol, and beverages, including caffeine. While consuming caffeine is generally considered safe during pregnancy, there is still much to learn about its impact on fetal development. As highlighted in this review, current clinical evidence shows maternal caffeine consumption can increase the fetus’s breathing rate and awake time. Additionally, it can raise the fetal heart rate and lead to a lower birth weight, which can impact the baby’s overall growth. Although no conclusive evidence links caffeine to gestational length or hypertension, it can increase uterine contractions, potentially resulting in spontaneous abortion or pre-eclampsia. However, it is unclear whether caffeine consumption increases the risk of congenital malformations. Therefore, further clinical trials with larger sample sizes are needed to investigate the effects of caffeine on congenital disabilities in infants. These studies should include patients with fewer confounding factors, such as smoking, alcohol use, medication, or recreational drug use. Such research endeavors will substantiate existing, limited evidence. Additionally, it is essential to understand the underlying mechanism through which caffeine affects fetal development and pregnancy. Such research would greatly benefit expecting women and their healthcare providers, ensuring a healthy pregnancy and reducing the likelihood of adverse fetal outcomes.
